# Supplementary material for: Comprehensive Immune Profiling Unveils a Subset of Leiomyosarcoma with “Hot” Tumor Immune Microenvironment
Source: Cancers (Basel). 2023 Jul 21;15(14):3705. doi: 10.3390/cancers15143705 (PMC10378143; doi:10.3390/cancers15143705)

Supplemental Table 1: **A:** Immune “hot” clusters T-H (identified by 90 gene signature method) and **B:** T-Hi (identified by CIBERSORT method) were strongly associated with Sarcoma Immune high Subclass E (SIC E) in TCGA cohort.

A

	T-C	T-H	p values
All Subjects	36	18	
SIC			<0.001
A	12(33%)	0(0%)	
B	13(36%)	2(11%)	
C	4(11%)	0(0%)	
D	7(19%)	6(33%)	
E	0(0%)	10(56%)	
NA	0(0%)	0(0%)	

90 gene signature

B

	T-Hi	T-L	T-M	p values
All Subjects	14	11	29	
SIC				0.094
A	1(7%)	5(45%)	6(21%)	
B	3(21%)	4(36%)	8(28%)	
C	0(0%)	0(0%)	4(14%)	
D	4(29%)	2(18%)	7(24%)	
E	6(43%)	0(0%)	4(14%)	
NA	0(0%)	0(0%)	0(0%)	

CIBERSORT

Supplemental Table 2: The correlation between immune clusters and clinical factors in ICGC cohort. Although, there was no association observed between immune clusters defined by 90 gene signature method and clinical factors **(A)**, there may be some association between immune clusters defined by CIBERSORT method and clinical factors **(B)**. For example, the immune “hot” cluster T-Hi seemed to associate with grade 3 in TCGA cohort indicated by red arrow.

A

	I-C	I-H	p values
All Subjects	38	49	
**Age.at.diagnosis**	67.5(29-83)	64(29-83)	0.235
**Sex**			0.359
Female	28(74%)	31(63%)	
Male	10(26%)	18(37%)	
**Size**	80(20-205)	80(15-230)	0.837
**Size.cut**			0.952
[15, 21)	1(3%)	2(4%)	
[21, 51)	10(26%)	11(22%)	
[51,100)	14(37%)	17(35%)	
[100,230]	13(34%)	19(39%)	
**Grade**			0.824
1	4(11%)	6(12%)	
2	19(50%)	21(43%)	
3	15(39%)	21(43%)	
**LMS Classifiers**			0.148
hLMS	20(53%)	16(33%)	
oLMS	6(16%)	14(29%)	
unclass	12(32%)	19(39%)	
**Location.of.first.metastasis**			0.958
Lung only	7(18%)	9(18%)	
No Met	20(53%)	24(49%)	
Other Met	11(29%)	16(33%)	
Site.of.tumour			0.34
Gyneacological area	5(13%)	3(6%)	
Internal trunk	19(50%)	23(47%)	
Others	6(16%)	15(31%)	
Upper.Lower.limb	8(21%)	8(16%)	
**Site.of.tumour.**			0.289
Gyneacological area	5(13%)	3(6%)	
Others	33(87%)	46(94%)	

B

	I-Hi	I-L	I-M	p values
All Subjects	16	54	17	
**Age.at.diagnosis**	64.5(60-75)	64.5(29-83)	64(43-83)	0.792
**Sex**				0.001
Female	13(81%)	41(76%)	5(29%)	
Male	3(19%)	13(24%)	12(71%)	
**Size**	80(30-170)	90(15-230)	70(30-205)	0.966
**Size.cut**				0.692
[15, 21)	0(0%)	3(6%)	0(0%)	
[21, 51)	5(31%)	13(24%)	3(18%)	
[51,100)	4(25%)	18(33%)	9(53%)	
[100,230]	7(44%)	20(37%)	5(29%)	
**Grade**				0.001
1	2(12%)	8(15%)	0(0%)	
2	4(25%)	32(59%)	4(24%)	
3	9(56%) ←	14(26%)	13(76%)	
**Location.of.first.metastasis**				0.493
Lung only	2(12%)	9(17%)	5(29%)	
No Met	7(44%)	28(52%)	9(53%)	
Other Met	7(44%)	17(31%)	3(18%)	
**Site.of.tumour**				0
Gyneacological area	4(25%)	3(6%)	1(6%)	
Internal trunk	4(25%)	36(67%)	2(12%)	
Others	8(50%)	7(13%)	6(35%)	
Upper.Lower.limb	0(0%)	8(15%)	8(47%)	
**Site.of.tumour**				0.072
Gyneacological area	4(25%)	3(6%)	1(6%)	
Others	12(75%)	51(94%)	16(94%)	

Supplemental Table 3: The correlation between immune clusters and clinical factors in TCGA cohort. Although, there was no association observed between immune clusters defined by 90 gene signature method and clinical factors **(A)**, there may be some association between immune clusters defined by CIBERSORT method and clinical factors **(B)**. For example, the immune "hot" cluster T-Hi seemed to associate with iLMS in TCGA cohort indicated by red arrow.

A

	T-C	T-H	p values
All Subjects	36	18	
**Age.at.diagnosis**	63(37-90)	58(33-82)	0.393
**Sex**			0.083
FEMALE	22(61%)	6(33%)	
MALE	14(39%)	12(67%)	
**Grade**			0.579
1	1(3%)	1(6%)	
2	26(72%)	11(61%)	
3	9(25%)	6(33%)	
**LMS Classifier**			0.662
hLMS	21(58%)	9(50%)	
oLMS	9(25%)	7(39%)	
unclass	6(17%)	2(11%)	
**HRD Score**	29(6-72)	20.5(0-61)	0.635
**LMS Subtypes**			0.303
cLMS	21(58%)	9(50%)	
iLMS	7(19%)	7(39%)	
**Location**			1
Internal trunk	21(58%)	11(61%)	
Limb	15(42%)	7(39%)	

B

	T-Hi	T-L	T-M	p values
All Subjects	14	11	29	
**Age.at.diagnosis**	58(42-80)	69(47-82)	63(33-90)	0.211
**Sex**				0.134
FEMALE	4(29%)	7(64%)	17(59%)	
MALE	10(71%)	4(36%)	12(41%)	
**Grade**				0.137
1	1(7%)	0(0%)	1(3%)	
2	9(64%)	5(45%)	23(79%)	
3	4(29%)	6(55%)	5(17%)	
**HRD Score**	32(0-61)	31(9-55)	20(5-72)	0.213
**LMS Subtypes**				0
cLMS	4(29%)	2(18%)	24(83%)	
iLMS	6(43%) ←	4(36%)	4(14%)	
**Location**				0.001
Internal trunk	7(50%)	2(18%)	23(79%)	
Limb	7(50%)	9(82%)	6(21%)	

Supplemental Figure 1. The dendrograms (generated from 90 gene signature method) demonstrated that immune hot signature **I-H (above)** and immune cold signature **I-L (below)** are mostly homogenous across different tumor regions of the same patient' tumor in ICGC cohort. There were 22 patients who had multiple tumor samples (between 2 -6) taken from the same tumor.

I-H

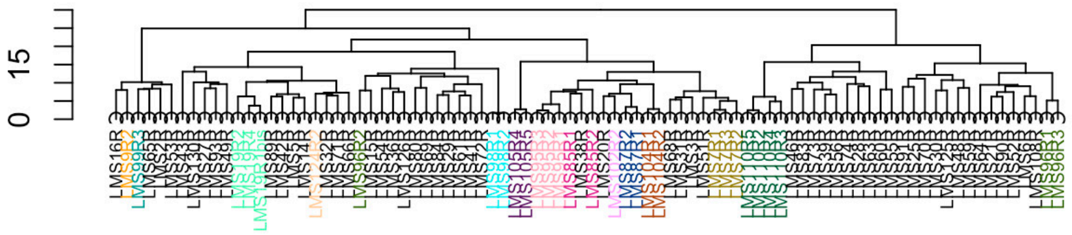

I-L

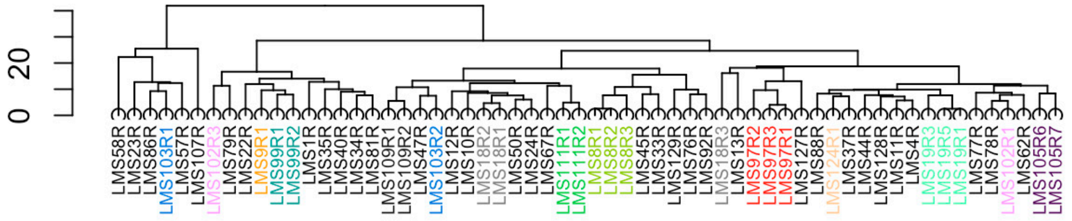

Supplemental Figure 2: Kaplan-Meier (KM) curves demonstrated that immune clusters regardless of methods (90 gene signature and CIBERSORT) used did not predict distant-metastasis free survival (DMFS) **(A and C)** as well as overall survival (OS) **(B and D)** in ICGC cohort.

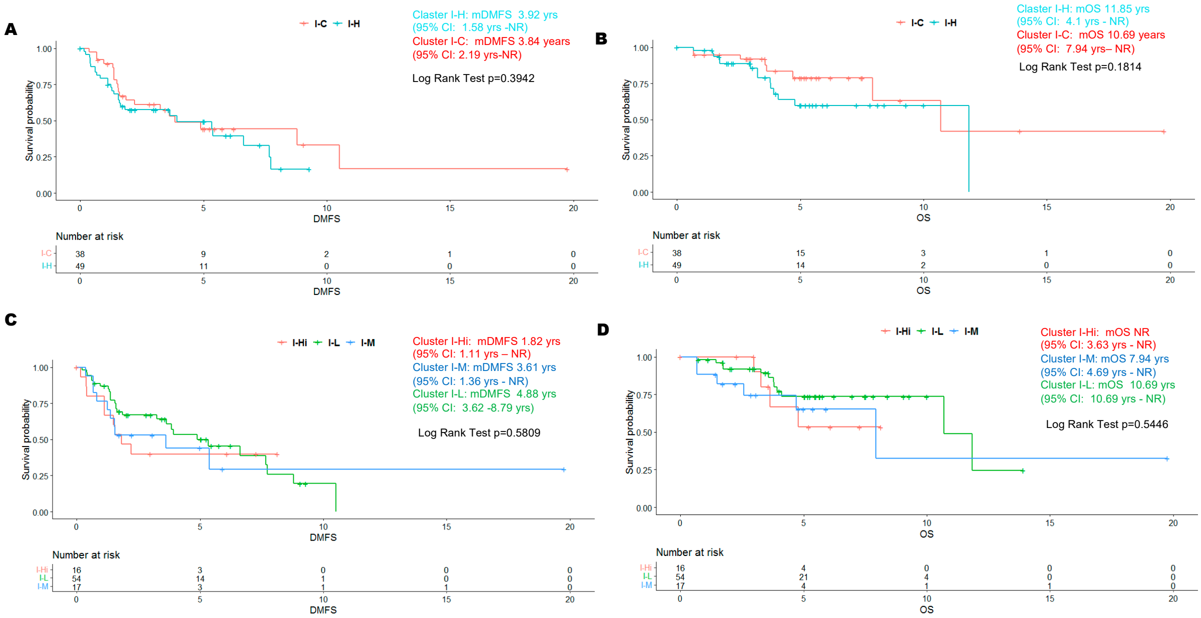

Supplemental Figure 3: Kaplan-Meier (KM) curves demonstrated that immune clusters regardless of methods (90 gene signature and CIBERSORT) used did not predict distant-metastasis free survival (DMFS) **(A and C)** as well as overall survival (OS) **(B and D)** in TCGA cohort.

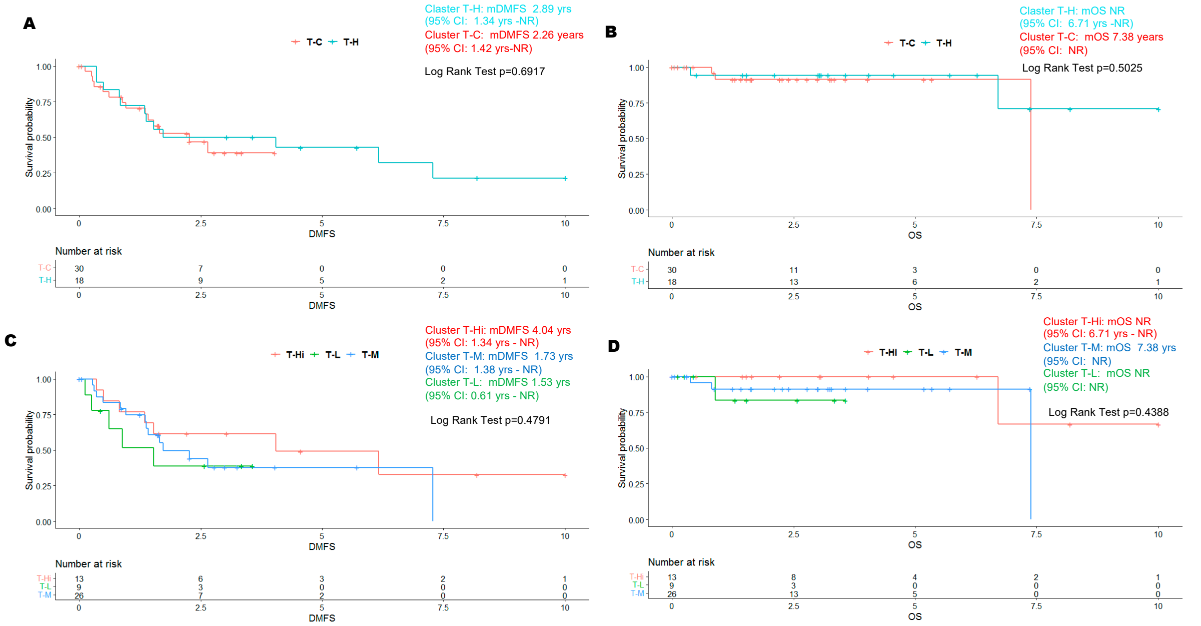

Supplement: Supplementary file 1 [file cancers-15-03705-s001.zip › cancers-2494501-supplementary.pdf]
